# Changes in Tetrodotoxin-Resistant C-Fibre Activity during Fatiguing Isometric Contractions in the Rat

**DOI:** 10.1371/journal.pone.0073980

**Published:** 2013-09-05

**Authors:** Ivana Kalezic, Heinz Steffens

**Affiliations:** 1 Department of Surgical and Perioperative Science, Sport Medicine Unit, Umeå University, Umeå, Sweden; 2 Centre for Musculoskeletal Research, University of Gävle, Umeå, Sweden; 3 Institute of Physiology, University of Göttingen, Göttingen, Germany; Emory University, United States of America

## Abstract

It is by now well established that tetrodotoxin-resistant (TTX-R) afferent fibres from muscle in the rat exhibit a multisensitive profile, including nociception. TTX-R afferent fibres play an important role in motor control, via spinal and supraspinal loops, but their activation and function during muscle exercise and fatigue are still unknown. Therefore, the specific effect of isometric fatiguing muscle contraction on the responsiveness of TTX-R C-fibres has been investigated in this study. To quantify the TTX-R afferent input we recorded the cord dorsum potential (CDP), which is the result of the electrical fields set up within the spinal cord by the depolarisation of the interneurons located in the dorsal horn, activated by an incoming volley of TTX-R muscle afferents. The changes in TTX-R CDP size before, during and after fatiguing electrical stimulation of the gastrocnemius-soleus (GS) muscle have been taken as a measure of TTX-R C-unit activation. At the end of the fatiguing protocol, following an exponential drop in force, TTX-R CDP area decreased in the majority of trials (9/14) to 0.75±0.03% (mean ± SEM) of the pre-fatigue value. Recovery to the control size of the TTX-R CDP was incomplete after 10 min. Furthermore, fatiguing trials could sensitise a fraction of the TTX-R C-fibres responding to muscle pinch. The results suggest a long-lasting activation of the TTX-R muscle afferents after fatiguing stimulation. The role of this behaviour in chronic muscle fatigue in connection with pain development is discussed. Accumulation of metabolites released into the interstitium during fatiguing stimulation might be one of the reasons underlying the C-fibres’ long-lasting activation**.**

## Background

It is by now well established that muscle fatigue and related pain syndromes accompany powerful and long-lasting activation of high-threshold muscle afferents [1]–[14]. This activation evokes complex effects in the central nervous system (CNS). Firstly, it provides the CNS with nociceptive information from the muscle. Secondly, it plays a substantial role in the development of adaptive segmental motor reactions, through rearrangement of complex spinal pre-motoneuronal network, recently mapped using c-Fos neuronal activity labelling in rats [9] and cats [15]. Thirdly, it participates in the reflex control of exercise-induced cardiovascular and respiratory adaptations [9]; [16]–[18].

The heterogeneous family of small-diameter afferent fibres embraces a particular fraction of fibres expressing a high density of tetrodotoxin-resistant (TTX-R) voltage-gated sodium channels (VGSC) with a conduction velocity predominantly within the C-fibre range [19]; [20], see also [21]–[23]. The projection zone of TTX-R gastrocnemius–soleus (GS) afferent fibres in the rat spinal cord has been described recently, the synaptic field potential of the TTX-R muscle afferents reached their maximum value in lamina IV-VI of the dorsal horn [24]. The functional role of TTX-R C-fibres has been examined in the cat, where TTX-R C-fibres contribute to the nociceptive reflexes of a flexor reflex afferents (FRA) pattern and of a non-FRA pattern [25]. However, the question of the modality of TTX-R muscle afferents is still under discussion.

Recent studies have provided novel information regarding the modality of stimuli to which these fibres may respond. In the rat, TTX-R afferent fibres respond to pinch and heat within the noxious range [20]. VGSC-Na_v_1.8 (which give rise to the TTX–R inward sodium current) is shown to be substantial for pain sensation at low temperatures [26]. Furthermore, the deletion of the α-subunit of the VGSC-Na_v_1.8 in mice results in stimulus-dependent deficit in the dorsal horn neurons encoding the mechanical input [27]. This finding indicates a predominant involvement of VGSC-Na_v_1.8 and somatic TTX-R currents in noxious mechano- and thermo-sensation in rodents.

The majority of the TTX-R fibres of the GS nerve activate interneurons of laminae IV to VI, as well as the surface laminae (I-III) of the corresponding segments [24], i.e. their input impacts spinal motor function, as well as nociception. In our study, we used the cord dorsum potential (CDP), which is a measurable sign of activation of mapped interneurons by TTX-R incoming volley from the GS muscle, to estimate the TTX-R afferent input quantitatively. While all fibres but TTX-R fibres were blocked by TTX, the size of the TTX-R CDP was taken as a measure of the TTX-R C-unit activation when electrically stimulated. It has been previously shown that an additional natural input (i.e., irregular non-synchronised input evoked by mechanical or thermal stimulation) through TTX-R C-fibres affects the TTX-R CDP [20].

Fatiguing stimulation results in C-fibre activation of the fatigued muscle [9]–[12]; [28]–[30]. However, as previously shown, afferent C-fibres are not a homogeneous class of fibres, there are different modalities in this class expressing subtypes of Na_v_ channels with different sensitivity to TTX. The faster fraction of all afferent fibres with conduction velocities of about 1 m·s^−1^ loses conductivity with TTX application ([19], cf. also [24], where peaks of the TTX-R synaptic field potentials are delayed). However, it remains unclear whether the C-fibres activated by fatiguing stimulation are sensitive to TTX or not. If electrical stimulation in the periphery evoked a maximum TTX-R CDP at the spinal cord, then this potential should be reduced when the TTX-R fibres are active at the same time, due to perception at their endings. Such behaviour may be due to the fact that a fraction of the C-fibres activated by natural input cannot respond to the electrical stimulation because of the refractory period. This phenomenon may be used to further classify the modality of the C-fibres that are activated by fatiguing stimulation (FST).

In this study, we assessed the responsiveness of TTX-R C-fibres to long-lasting muscle contraction by recording the TTX-R CDP during and following fatiguing trials to answer the question whether long-lasting muscle activity modulates TTX-R afferent inflow and to what extent muscle fatigue affects the sensitisation of muscle nociceptors.

## Materials and Methods

The experiments were performed on 11 male Sprague-Dawley rats weighing between 250 and 380 g. The animals were purchased from state-controlled animal farms via the common animal facility of the University of Umeå. The experiments were performed strictly according to the NIH guidelines for the use of experimental animals and with the approval of the local Ethics Committee (Umea Djuförsöketiska Nämnd, Proj A 114-01).

The animals were housed in groups of 3 in plastic cages where the floor was covered with soft sawdust bedding under a 12 h light/12 h dark cycle (lights on 06∶00 h). The animals were kept in a room at a constant ambient temperature, and water and food were provided *ad libitum*. The rats were habituated for at least seven days for experimental manipulation. All efforts were made to minimise animal suffering during the experiments. Two animals died during the experiment, and the others were sacrificed at the end of the experiment.

The method of preparation, eliciting and recording of the TTX-R CDP were performed as previously described [19]; [20]. The rats were initially anaesthetised with an intraperitoneal injection of 80 mg/kg pentobarbital sodium (Nesdonal, Apotekets produktion och laboratoria, Umeå). A catheter was inserted into the right external jugular vein for further continuous administration of anaesthetics (approximately 20 mg kg^−1^ h^−1^ of Nesdonal) in 0.9% NaCl solution as needed to abolish the withdrawal reflex in response to pinching of the hind paw, as well as the corneal reflex and marked blood pressure reactions (exceeding 10 mm Hg) to noxious stimuli. The trachea was exposed and an endotracheal tube was inserted. The common carotid artery was cannulated for blood pressure measurement. Mean arterial blood pressure, heart rate and rectal temperature were continuously maintained at physiological levels (above 80 mm Hg, below 400 bpm, and at 37–38°C, respectively). Prior to the beginning of the virtual experiments, the rats were paralysed with pancuronium bromide (Pancuronium ‘Organon’, 2.5 mg/kg initially i.v. and further with 1.5 mg·kg^−1^·h^−1^) and were artificially ventilated by maintaining the expired CO_2_ at 3–4%.

**Figure 1:**
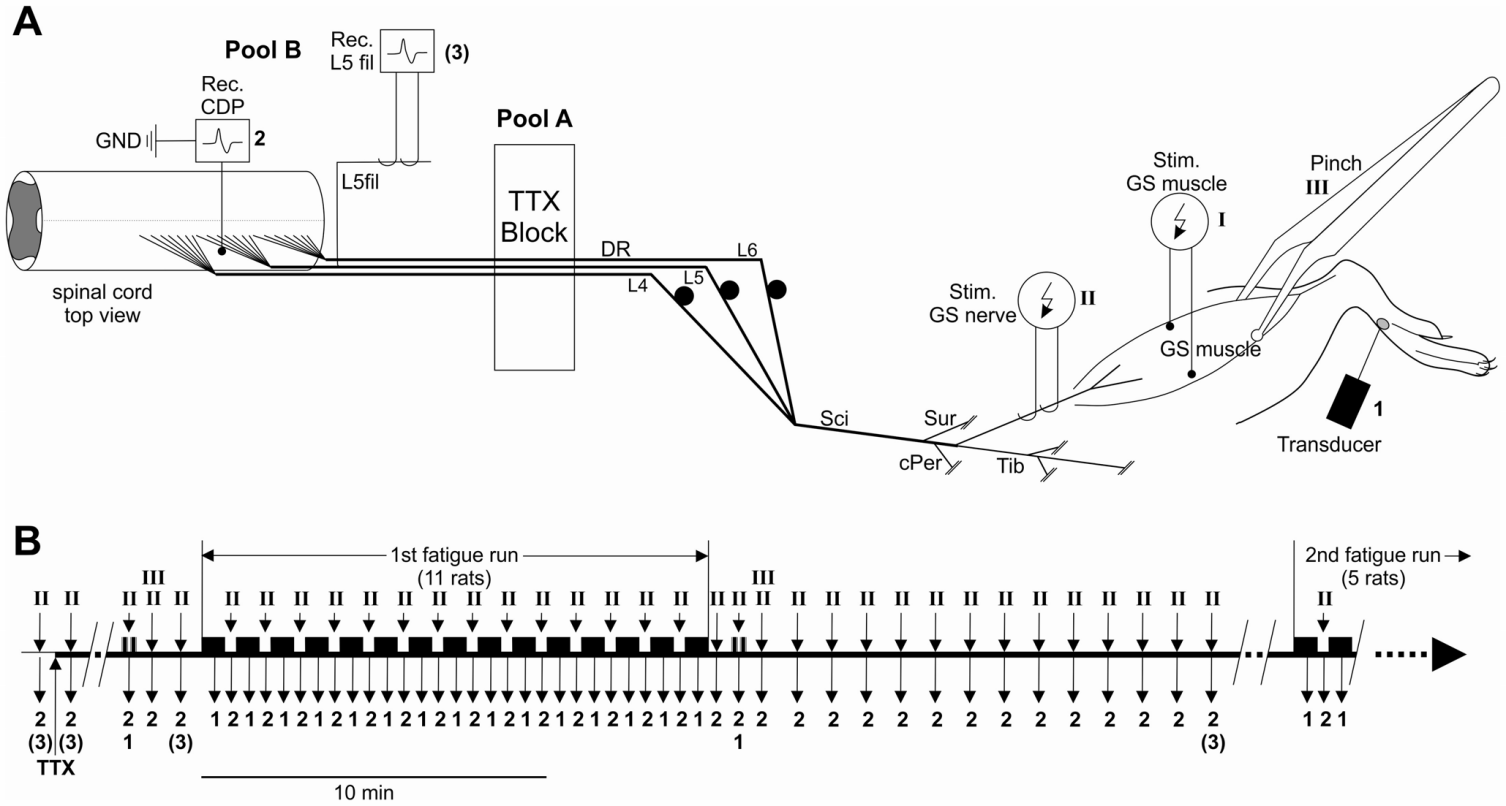
Experimental set-up (A) and stimulation protocol (B). Stimulation sites are marked by Roman numerals, recording sites are marked by Arabic numerals. Stimulation (Stim.) sites (A) at the GS muscle (I, electrical; III, pinch) and GS nerve (II); recording (Rec.) sites at the foot (1, Force transducer), dorsal root entry zone of dorsal roots L4–L6 (2, Rec. CDP), and filament of dorsal root L5 (3, Rec. L5fil). Recording #3 is set in brackets because it is not depicted in this publication. The sciatic (Sci) and the gastrocnemius-soleus (GS) nerve were left intact while the sural (Sur), common peroneal (cPer), and the rest of the tibial (Tib) nerves were transected. B shows the complete fatiguing stimulation pattern with preceding control tests and TTX application and the following rest period with the test stimulation; electrical stimulation of the GS muscle represented as bars (▪). In five out of eleven experiments, a second fatiguing stimulation run was applied after at least a 30-min rest period between the end of the first fatigue run and the beginning of the second fatigue run. Only the beginning of the second stimulation and recording cycle are displayed.

Laminectomy was performed to expose the dorsal roots L4–L6 in full length (dorsal root L5 contains most but not all of the lower leg afferent input). The animals were secured within a frame (Kopf instruments) using clamps that were fixed onto the vertebra rostral and caudal of the laminectomy (L1 and S1, respectively). The dorsal root L3 and ventral roots L3–L6 were cut to avoid ortho- and antidromic A-fibre input via these roots during electrical stimulation of the nerves at C-fibre strength (for experimental set-up cf. Figure 1A).

Underneath the intact dorsal roots L4–L6 on the left-hand side, a piece of expanded laboratory film (Parafilm) measuring 8–10 mm in width was placed rostral of the dorsal root ganglion L4. The exposed spinal segments and dorsal roots were then covered with boiled 3% agar in Ringer’s solution at 38°C. Two pools were cut in the agar: one above the Parafilm (pool A), which was approximately 5 mm wide, and another which was placed more rostral (pool B) to expose the spinal segments L4–L6 together with approximately 10 mm of dorsal roots (DRs) L5 near their entrance into the spinal cord. Pool B was filled with warm mineral oil. Pool A was filled with warm Ringer’s solution, and served later for TTX application.

The nerves of the gastrocnemius-soleus (GS) muscle of the left hind leg were exposed, left in continuity and subsequently mounted together on a hooked bipolar platinum wire electrode for stimulation. All of the other nerves (cutaneous and muscle nerves) were transected. The belly of the GS muscle was also exposed in full length. A pool was formed by the skin flaps of the left hind leg, and the exposed GS nerves and muscle were covered with warm silicon oil. The temperature of the pools over the exposed spinal cord and at the left hind leg was maintained close to 37°C using radiant heat.

The force transducer was coupled to the dorsum of the foot, while the GS tendon was left intact at its distal insertion in order to avoid tying it to the force transducer and thereby evoking C-fibre input.

Platinum ball electrodes positioned on the dorsal surface of the spinal cord at the L4–L6 dorsal root entry zone were used for unipolar recording of the cord dorsum potentials (CDPs) against ground. Occasionally, an L5 rootlet was cut centrally and split, and the spontaneous activity in the dorsal root filaments of the L5 segment was recorded to assess the TTX-evoked abolition of spontaneous activity from the TTX-sensitive afferent fibres.

**Figure 2:**
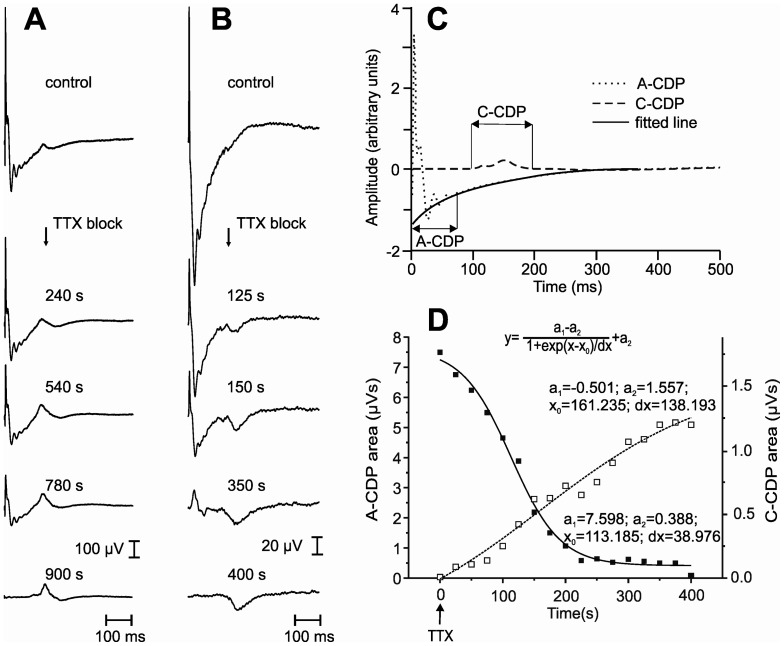
Changes in the CDP evoked by electrical stimulation of the GS nerve at C-fibre strength following TTX application on dorsal roots L4–L6. Two examples (A, B), which display the C-CDP without TTX and the development of the TTX block after TTX application are given. The recordings were performed in two rats. The C-CDPs appeared at ∼128 ms latency. (A) The C-CDP response became more prominent while the A-CDP diminished and vanished. (B) The C-CDPs appeared just after the TTX application, but were otherwise not noticeable. A latency shift of the C-fibre-evoked CDP could also be observed. (C) Multiple linear regression of the form 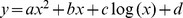 was fitted to the ascending part of A-fibre CDP trajectory, which allowed for a clear distinction between the A-CDP and C-CDP envelopes and furthermore, a selective estimation of the A- and C-CDP area. (D) A sigmoidal approximation might satisfactorily describe the changes in A-CDP and C-CDP areas induced by the application of TTX (areas taken from experiment shown in B).

The gastrocnemius nerve was stimulated at a constant voltage (25–50 V, pulses of 0.3 ms duration at 3-s intervals) in order to evoke C-fibre effects (position of the electrodes cf. Fig. 1A). The stimulation strength was adjusted in relation to the lowest threshold of the A-fibres, where we found thresholds of 150 mV to 300 mV at 0.1 ms stimulus duration; thus, the applied stimulation strength was well above the threshold for C-fibres, which is 50 to 100 times the threshold of the A-fibres for the corresponding nerve. After determined the electrical stimulation threshold of the GS nerve, Ringer’s solution of pool A was replaced with 1 µM TTX in Ringer’s solution in order to block the dorsal roots L4–L6 completely, except for the TTX-R C-fibres (cf. [19], also Fig. 2).

**Figure 3:**
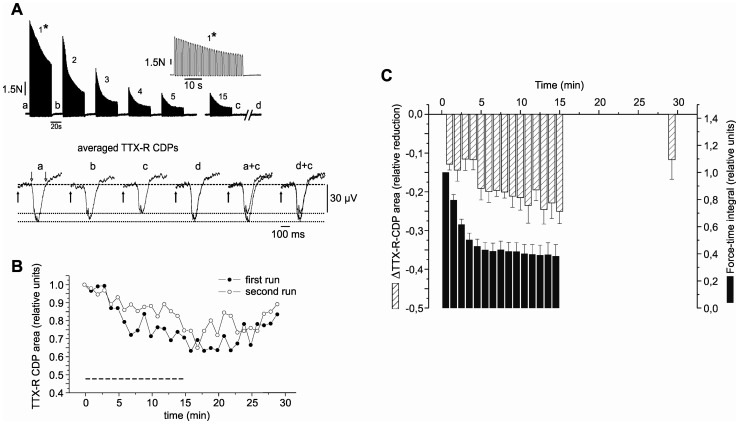
TTX-R CDPs during 15 min of fatiguing isometric contractions of the GS muscle. (A) Muscle contractions during the first five and the last (15th) stimulation session, and corresponding averages of the 6 individual filtered (time constant 0.001 s) TTX-R CDPs before the beginning of the FST (*a*), during the 20-s period of rest between the successive contraction trials (*b*), after the end of the FST (*c*), and at the end of the recovery period (*d*). The responses were evoked by a single stimulus (↑) of a 0.3-ms duration with a rate of 0.3 Hz, at 2.27 times above the threshold for appearance of the C-CDPs. Note the reduction of TTX-R CDPs at the end of the FST compared to the control (*c* vs. *a*) and the recovery afterwards (*c* vs. *d*). The 40-s periods of intermittent stimulation were separated by 20 s of rest. The first force response to the muscle stimulation period (_*_) is also presented at a higher time resolution scale. Each train in a session of intermittent stimulation consisted of regular impulse trains at 100 Hz and with a 500-ms duration, separated by 500-ms intervals of rest. Open down arrows in *a* indicate integration time for area measurements. (B) Time dynamic of changes in TTX-R CDP during the fatiguing contractions and its gradual restoration from an individual experiment in which the stimulation protocol was applied twice. For every minute, 6 CDPs were recorded either during the FST intervals or during the 15 minutes of rest. The areas of the 6 CDPs were integrated over the time interval as indicated by the down-arrows in Fig. 3Aa, normalised to the mean pre-FST value of the TTX-R CDP area and plotted as a function of time. In the second runs, the depression of the TTX-R CDPs, as well as their restoration after FST did not differ in principle from the reactions observed during the first FST. The FST period is marked by a bar below the time courses of the CDP area. Same experiment as (A). (C) Summary of the quantitative changes in the TTX-R CDP and force (mean ± SEM) during and after 14 FSTs (11 experiments, three 2nd FSTs). A relative reduction in the TTX-R CDP was calculated as 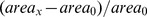. In 9 FST stimulation cycles, the recovery of the TTX-R CDP was only randomly tested to allow the preparation a substantial rest; therefore, the columns were omitted between 15 min and 29 min.

Long-lasting muscle contraction and fatigue were evoked using direct current stimulation (pulse duration 0.2 ms) of the GS muscle (stimulation protocol cf. Fig. 1B). The stimulation current was set to three to five times the motor threshold, i.e. the range of the applied stimulus currents varied in different experiments between 1.5 and 2 mA, which was always lower than the threshold for the electrical activation of group III and IV afferents in the hind limb nerves in rat [9]; [31] but evoked a maximum twitch of the GS muscle. The spinal effects of the low-threshold GS muscle afferents, which were recruited by electrical stimulation and/or twitch of the muscle, were completely abolished after the TTX block. The protocol for evoking high-frequency fatigue was as follows (Fig. 1B, cf. also Fig. 3A): the entire duration of the 15 min fatiguing stimulation was divided into repetitive one-minute periods. Each minute was partitioned into a 40-s period of intermittent stimulation and a 20-s rest period. The 40-s stimulation period was divided into repetitive one-second intervals, which consisted of 500 ms of regular stimuli at 100 Hz and 500 ms of rest. After a 30-min rest period, five out of eleven rats underwent a second fatiguing stimulation run of 15 min (cf. Fig. 1B), where two of the rats died during the second fatiguing stimulation. The muscle was considered to have recovered when the tension changes that were evoked by the stimulation applied after the period of rest, were the same as that prior to the stimulation session.

Instead of force, the torque was measured by connecting the dorsum of the foot at a distance of approximately one-third to half of the entire foot length from the ankle joint to the force transducer using a thread. The compliance of the force transducer did not exceed 50 µm N^−1^ and its resonant frequency was close to 1.5 kHz. Since contractions were performed under isometric conditions, and the distance from the selected point to the centre of rotation (ankle joint) remained constant throughout consecutive muscle contractions, the measured changes in torque were proportional to the changes in the GS force.

**Figure 4:**
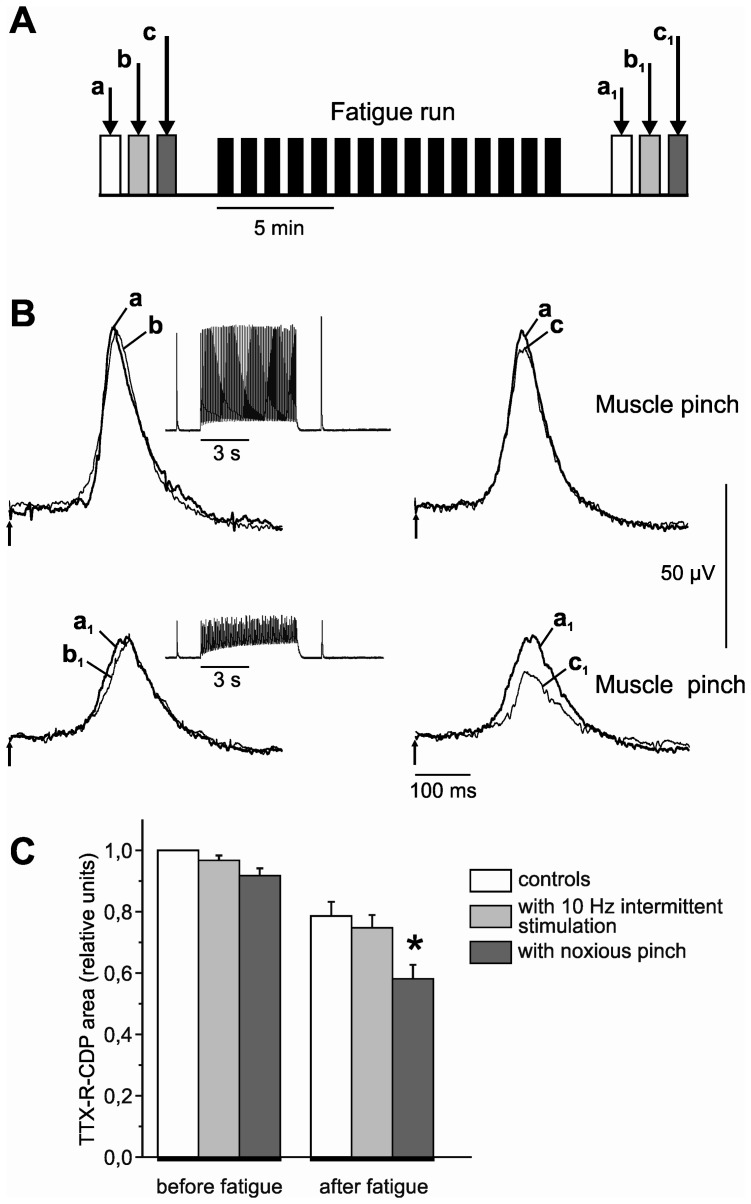
Sensitisation of muscle nociceptors by FST. (A) experimental protocol. (B) CDPs evoked by electrical stimulation of the GS nerve at 25 V (recurrent frequency 0.3 Hz; duration of stimulus 0.3 ms) during the TTX block of the L4–L6 dorsal roots. Control responses before the FST (B, first row, a, b, c) compared to the responses after the FST (B, second row, a_1_,b_1_,c_1_). a and a_1_ control CDPs without additional stimulation; b and b_1_ CDP recordings during two sessions (cf. Fig. 1B) of low-frequency intermittent muscle stimulation (10 Hz train of a 6 s duration, preceded and followed by single twitches, 5 s intervals between the successive sessions, cf. inserts of force response), c and c_1_ CDP records during muscle belly pinching by flat forceps for approximately 30 s. Averaged responses, n = 6. Trigger impulse for the electrical stimulation is depicted by the arrow pointing upwards. Pinching of the muscle belly evoked a larger reduction in the TTX-R CDPs after the FST compared to control, while the changes in the low-frequency stimulation were absent in both cases. (C) Comparison of the test CDPs before and after fatigue for n = 5 experiments (mean ± SEM). The column marked by an asterisk is significantly different (p<0.05) to the corresponding column before fatigue.

In five experiments the effect of the isometric muscle twitches and the noxious stimulation of the GS muscle (pinch using flat forceps) on the TTX-R CDP was tested before and after the first fatiguing run (for protocol see Fig. 4).

### Data Acquisition and Analysis

The CDPs, muscle tension and blood pressure were sampled using the CED Power 1401 (Cambridge Electronic Design (CED™), UK) while the Spike 2 software (CED) was used for data acquisition and further processing. The input signals were digitised with 12-bit resolution at rates of 1 kHz (blood pressure and muscle force) and 10 kHz (CDPs). The data analysis was performed using the Origin 7.0 software (OriginLab™ Corporation, USA). Statistical analysis of the changes in CDPs during and after muscle fatigue was conducted using repeated-measures analysis of variance (ANOVA). Provided that changes in the studied parameter were significant in successive time intervals, post hoc comparisons were performed using the Bonferroni adjustment for multiple comparisons. The statistical analysis of changes in the CDPs evoked by muscle twitch/pinch before and after the conditioning stimulation was performed using one-way ANOVA. Differences were considered significant at p<0.05. Statistical analyses were performed using SPSS 10.0 for Windows (SPSS Inc. (IBM™), USA).

## Results

### Effects of TTX Blockade

When the GS nerve was stimulated with a strength well within the group C range, C-fibre evoked CDPs could be observed in approximately 50% of all experimental trials (a representative example is shown in Fig. 2A) before the application of TTX. After the application of TTX, the C-fibre evoked CDP was evident in all cases (representative examples are shown in Fig. 2A and B, each panel shows traces of averaged CDPs (n = 6) recorded from the two experiments). The distance between the stimulation and recording electrodes was 110 mm, and the calculated conduction velocity of the C-fibres was 1 m·s^−1^ or less. These findings confirmed a similar previous observation [20]. Following TTX application, the changes in the C-CDPs occurred in parallel with the diminishment of the A-fibre effects. These changes in the potentials could be observed in all of the experiments. A decrease in the A-fibre CDPs was usually followed by an increase or appearance of the C-CDPs (Fig. 2). A latency shift of the C-fibre evoked CDP was also observed. Time dynamics of the TTX-blocking effects were quantified using multiple regression analyses, where 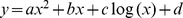 was fitted to the ascending part of the A-fibre CDP trajectory. Such an approach enabled a clear distinction between the A-CDP and C-CDP envelopes (Fig. 2C) and further selective estimation of the A- and C-CDP area. A sigmoidal approximation as shown in Fig. 2D might satisfactorily describe the changes in CDP areas, appearing at A- and C-fibre latencies (reproduced from Fig. 2B). In this experiment, the estimated C-CDP area before the TTX block was just visible (0.0075 µVs), but subsequently became markedly enlarged (1.1975 µVs).

Spontaneous A-fibre activity, recorded from an occasionally isolated and centrally sectioned afferent filament (cf. Fig. 1A, records not shown) vanished together with the A-CDP (previously shown, see [19]).

### Changes in the Tetrodotoxin-resistant C-fibre Afferent Input by Long-lasting Muscle Contraction

When the TTX block was shown to be complete, i.e. when no more effects with latencies corresponding to the conduction velocities faster than 1 m·s^−1^ were detected, we assessed whether muscle fatigue might affect TTX-R afferent inflow into the spinal cord. In order to avoid possible effects of muscle nerve displacement induced by strong tetanic muscle activation on the recorded potentials, changes in the TTX-R CDPs were recorded immediately after the cessation of successive contraction trials (i.e. during 20-s period of rest) [9]; [10]. Fig. 3A shows traces of the averaged records (n = 6) between the successive contraction trials labelled as: *a* (control period), *b* (after the first tetanic session), *c* and *d* (periods at the end of FST and 10 min afterwards, respectively). When compared to control recordings, the TTX-R CDPs at the end of FST (Fig. 3A, *a* and *c* superimposed) were markedly reduced. CDPs could also slightly decrease following the first contraction run (Fig. 3A, cf. *b* and *a*). A partial recovery of the TTX-R CDP was observed (Fig. 3A, *d* and *c* superimposed). For each particular experiment, the averaged CDP recordings of the successive test periods were integrated over time, as indicated by the open down arrows in Fig. 3Aa. These values were normalised to the mean pre-test value of the TTX-R CDP area at rest (determined in a 60–90-s period preceding the onset of the FST) and were plotted as a function of time. In five experiments, the FST protocol was repeated twice. In the second runs, the depression of the TTX-R CDPs, as well as their subsequent restoration, did not differ from the responses initially observed on the fresh muscle. An example of such an experiment is presented in Fig. 3B. The reduction of the TTX-R CDP by FST could be observed in six first FST runs and three second FST runs, i.e. in nine out of fourteen FST runs. In the experiments where the animals died during the second run no changes in the TTX-R CDP were observed during the first FST; this was also observed in three out of the six experiments where only one FST run was performed. Nevertheless, the results of all the FSTs (n = 14) were pooled and plotted in Fig. 3C.

Fig. 3C shows the summary of quantitative changes in TTX-R CDPs during and after the FST in 11 experiments, three of which underwent a second FST run, in relation to force development during FST. Immediately after the end of the FST, the relative TTX-R-CDP area, following an exponential drop in force, decreased to 0.75±0.03 (mean ± SEM) of the pre-fatigue values. Relative reduction in the TTX-R CDP was calculated as 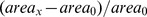. This reduction was significantly higher (p<0.05) than the reduction in TTX-R-CDPs, which was evoked by the first tetanic stimulation period during FST (0.87±0.02; mean ± SEM of the pre-fatigue values). Ten minutes following the end of the FST, CDPs partially recovered to 0.88±0.04 (mean ± SD) of the control values (this recovery reached statistically significant levels, p<0.05).

However, as previously mentioned, in certain cases (35.7% of all trials), the alterations in the TTX-R CDP area were completely absent or negligible. However, whenever the effect was present, in each of the experiments the TTX-R CDP area decreased.

In five experiments we addressed the question whether prolonged muscle activity could evoke sensitisation of TTX-R C-fibres (Fig. 4). As previously shown, TTX-R CDPs could decrease during painful pinching of the muscle belly [20]. In our experiments, noxious pinching of the GS muscle for 20–30 s caused a small but visible reduction in the TTX-R CDP (compare c and a, Fig. 4B); such responses were observed in 62.5% of all experiments which were conducted before and after the first FST run. Otherwise, TTX-R CDPs remained almost unchanged during small mechanical distortions of the C-fibre receptive field evoked by muscle twitches or short-term low-frequency (10 Hz) intermittent muscle contractions (compare b and a). After the fatiguing protocol, the TTX-R CDPs were markedly reduced (compare a and a_1_) and remained unchanged in response to low-frequency test contraction (compare a_1_ and b_1_) but were further reduced during painful pinching of the muscle belly (compare a_1_ and c_1_). In other words, after FST, the response of TTX-R C-fibres to mechanical, non-noxious stimuli remained unchanged, but the response to noxious stimuli could be potentiated. Due to noxious pinching of the muscle belly, the TTX-R CDPs were reduced to 0.91±0.02 (mean ± SEM, n = 5) of the control values, this reduction showing a trend towards statistical significance (p = 0.068). However, after FST, noxious pinch of the muscle caused a significant reduction in these potentials to 0.58±0.04 (mean ± SEM, n = 5) of the control responses (p<0.05).

## Discussion

### Properties of Tetrodotoxin-resistant Cord Dorsum Potential

When a constant TTX concentration (1 µM) was applied, the duration of the TTX-blocking procedure varied from 5 to 15 minutes in different animals (cf. [19]). These differences could be explained by various factors. The size of the TTX pool determines the effective concentration of TTX, since TTX also diffuses into the surrounding agar. Furthermore, the diameter of the dorsal root determines the time required for diffusion into the entire root.

Several reasons may justify the need to record the CDP, rather than to record the activity from the dorsal root directly: action potentials of the TTX-R C-fibres are small compared to the amplifier noise and therefore must be averaged even if recorded from a dorsal root filament (cf. [19]). To quantify the irregular TTX-R C-fibre input caused by the FST, a direct recording from the dorsal root appears inappropriate. Moreover, the space for a TTX pool and direct recording from the dorsal root is limited, if the dorsal roots are left intact, and TTX-R CDP impacts the direct measurement over large distance. Furthermore, the method provides hints about the dorsal horn circuitry. TTX-R CDP is the result of the electrical fields set up within the spinal cord by the depolarisation of interneurons located in the dorsal horn and activated by an incoming volley from TTX-R muscle afferents. The activated interneurons are mapped in laminae I to VI by Lambertz et al. [24]; [32] using synaptic field potential recording.

The shape of TTX-R CDPs in our experiments was not uniform. Accordingly, there are no evidence or literature data that C-fibres evoked CDP (particularly TTX-R CDP) always has a distinct shape, which enables clear discrimination of N and P wave in analogy to A-fibres evoked CDP [33]. The shape of the C-fibre evoked CDP is very much dependent on recording conditions, i.e., the exact position of the recording electrode and the ground electrode, the size and configurations of the electrodes, the quality of the contact to the cord dorsum. Especially the control of location is difficult, because local anatomy varies individually. In most cases, the L5 dorsal root carries the main input from the hind leg. Accordingly, dorsal root L5 is the largest among the lumbar spinal roots, however due to individual variability, the thickness of L4 and L5 may be equal. On the other hand, one may assume difficulty to obtain reproducible hypothetical P waves of C fibres evoked CDP (associated to primary afferent depolarization (PAD) and dorsal root potentials (DRPs) in analogy to A-fibres evoked CDP) due to large temporal dispersion of these potentials. Indeed, Steffens et al. [19] demonstrated that even in a small dorsal root filament the C-fibre response to peripheral electrical stimulation could already be dispersed over about 60 ms.

Willis [33] claimed that primary afferent depolarization and dorsal root potentials were demonstrated exclusively in thicker fibres down to Aδ-fibres. Part of recorded potential in our experiments might be PAD of myelinated afferents in the mapped region, but this does not influence the main finding, namely the changes of the central response to muscle fatigue. Described experimental manipulation, which included the TTX block and the transection of the ventral roots and dorsal root L3, was performed to ensure input exclusively from unmyelinated C-fibres.

A latency shift of the C-fibre evoked CDP, as found during the application of TTX to the C CDP of the cutaneous and GS muscle afferents [19]; [24] was also present in our experiments. Conduction velocity may only be estimated as an average for the total length of the distance from stimulation to recording, as the velocity of TTX-R C-fibres may change rostral to the TTX pool [19]. The increase of the C-fibre evoked CDP may be explained by the releasing from presynaptic inhibition, since the main projection region of the blocked A-fibres are spinal cord laminae IV to VI, the same region where the synaptic field potential (SFP) of GS C-fibre afferents was the most prominent [24]. TTX application also abolished activity from TTX-sensitive C-fibres and this effect probably contributed to reduction of CDP area; however such an event was negligible compared to effect evoked by release from presynaptic inhibition originating from A and TTX-sensitive fibres. Presynaptic inhibition of primary afferent terminals of C-fibres or the presynaptic inhibition of the first-order interneurons on which they impinge, originating from neighbouring C-fibres [34] is very well documented.

### Muscle Fatigue Induced Changes in TTX-R Afferent Inflow

The main aim of this study was to examine the effects of long-lasting fatiguing muscle contraction on tetrodotoxin-resistant C-fibre inflow into the spinal cord. It has been well established that fatiguing muscle stimulation activates a large spectra of small-diameter afferent fibres due to mechanical distortion of their receptive field and due to accumulation of metabolites and inflammatory substances in the intramuscular interstitial space [3]; [9]; [12]; [35]–[39]. Furthermore, muscle ischemia, maintained after the contraction, sustains the discharge of many small-diameter muscle afferents [40]. However, the responsiveness of the TTX-R C-fibres to the long-lasting, intermittent, isometric muscle activity remains unknown.

The size (area) of CDPs evoked by electrical stimulation of peripheral C-fibres from GS muscle in combination with natural stimulation of their receptive field was used for quantitative estimation of TTX-R afferent input. As TTX-R C-fibres were tonically active because of the effects of muscle contraction and fatigue, the probability of their response to electrical stimulation was concomitantly lowered. Thus, the reduction in CDP size was taken as a measure of TTX-R C-units activation.

Whenever the effect was present, fatiguing stimulation of the GS muscle evoked a reduction in the electrically evoked CDP (in 64.3% of trials). High frequency, long-lasting isometric muscle contractions in the majority of the cases enhanced the net TTX-R afferent inflow into the spinal cord. Immediately after the cessation of the FST, the mean TTX-R CDP area decreased to approximately 0.25 of the control value. This decrease was significantly higher than the reduction in TTX-R CDP area evoked by the first session of tetanic contraction. The TTX-R inflow at the beginning of contractions was rather small despite the submaximal-maximal forces of the GS muscle evoked by the high-stimulus frequency (100 Hz). This was surprising because of the presumed involvement of the TTX-R current in mechanosensation [27]. In our study time dynamics of the changes in the TTX-R CDP area reflected a prevailing response of the TTX-R C-fibre units to fatiguing effects, when other components, such as accumulation of metabolites and inflammatory substances in the intramuscular interstitial space and partly hypoxic condition might contribute to the tonic activation of the recorded fibres.

Nevertheless, this effect was smaller than expected because increases in the spontaneous discharge rate of single C-units as high as 50% and 200% compared to the baseline activity were reported to follow fatiguing rhythmic contractions of the diaphragm in rats [11] or the tibial muscle in rabbits [10], respectively. In addition, after long-lasting activity of the triceps-surae muscle, c-Fos immunoreactivity in the dorsal half of the spinal grey matter in the rat lumbar spinal cord was significantly enhanced [9]. The lamellar distribution of c-Fos labelled neurons overlapped almost completely with the projection zone of the high threshold muscle afferents [14].

Recently, Lambertz et al., 2008 demonstrated that the TTX-R spinal field potentials (SFP) were mainly reduced by myositis-induced TTX-R C-fibre activity. The direction of changes of the synaptic field potentials was highly dependent on the segment from which they were recorded. Indeed, in our work, the size of the CDPs was dependent upon the position of the electrode on the spinal cord, since spatio-temporal facilitation to activate the relevant interneurons changes along the spinal cord. Despite trying to position the electrode always at the same location on the spinal cord, we could not exclude that by anatomical individualities, the functionality of this location was different. Furthermore, it has already been shown that the position of the hip joint influences the spinal circuitry of interneurons and its function in motor control [41]. Despite having carefully attempted to consistently mount the animals in the same manner, with the leg and hip joints always in the same position and angle, we could not exclude small variations, which together with anatomical variations caused the reduction in the TTX-R CDPs area after FST in 55% of the animals. Nevertheless, it can be argued that if there was a change in the TTX-R CDP area, the CDP was reduced.

Several additional factors may also account for the moderate intensity of the observed effects or for their absence. Sub-maximal, isometric contractions provoked tonic activation of C-fibres, which did not elicit discrete compound action potentials but did evoke a more irregular activation of dorsal horn interneurons. Due to cross-activation, C-fibres within the common sciatic nerve might also be activated, thus contributing to the magnitude of the CDP area. However, only a small number of the TTX-R C-fibres were activated by repetitive tetanic contractions, and not all of the activated fibres had discharged at their maximal rate. Thus, taken together, this may lower the probability of the modulation of the electrically evoked TTX-R CDP. However, the number of excited TTX-R C-fibres was not the main determinant for the observed changes since it was suggested that the spinal effects of “small” TTX-R C-fibres of the sural nerve on the dorsal horn interneurons overwhelmed the spinal effects of large C-fibres [19].

Constant C-fibre input, evoked by electrical stimulation *per se* (albeit at a lower frequency), might evoke some changes in the excitability of first-order dorsal horn interneurons, i.e., it might create some pre-conditions for wind-up. However, an increase in the TTX-R CDP area as a consequence of cumulative depolarisation evoked by electrical stimulation was never observed; thus, the possibility that a wind-up occurred in our stimulus paradigm can be excluded.

It is assumed that the TTX-R C-fibres are intercalated in pain pathways [42]. Hyperalgesic mediators, such as prostaglandin E2 (PGE2), serotonin and adenosine, decrease the activation threshold, increase the rates of activation and inactivation and increase the magnitude of TTX-R Na^+^ currents [42]. The pronounced reduction in the CDP area, evoked by painful pinching of the muscle belly [20] following the FST, was significantly enhanced, indicating a sensitisation of the TTX-R fraction of nociceptors with a mechanosensitive profile by long-lasting fatiguing trials. Moreover, it also indicated that the FST failed to activate a major group of nociceptors with a mechanosensitive profile, suggesting that the modality profile activated by the FST was different from the one activated by the noxious pinch. Thus, sensitisation of the TTX-R fibres, together with tonic activation of the TTX-R muscle afferents, might be assumed to be a part of the mechanism connecting mechanical loading and chronic muscle pain. On the contrary, the C-CDP area remained almost unaffected by small mechanical distortions of the C-fibre receptive fields evoked by muscle twitches or short-term, low-frequency (10 Hz) muscle contraction. This result might suggest that, if present among the TTX-R C-fibres, non-nociceptive C-fibres were not susceptible to sensitisation provoked by muscle fatigue.

It has been demonstrated that presynaptic inhibition derived from group III and IV afferent fibres may play an important role in the restriction of afferent inflow to the CNS from the muscle spindles during the development of fatigue [12]. However, there is evidence that the activity of interneurons intercalated in the pathways responsible for nociceptive reflex activity is profoundly influenced by proprioceptive activity. A-fibres interact presynaptically with C-fibres to reduce their synaptic effectiveness [34]. Due to obvious methodological constraints, low incidence and a low signal-to-noise ratio of the TTX-R CDPs in the presence of the A-fibre input tracing of the time dynamic of presynaptic inhibition exerted from large- to small-diameter afferent fibres during and after fatigue was not achievable in this study.

## Conclusions

In this study, the particular effect of isometric fatiguing muscle contraction on the responsiveness of TTX-R C-fibre muscle afferents was explored. The results suggested a long-lasting activation of TTX-R muscle afferents after FST, similar to what has been shown for the activity of TTX-R afferents of the GS muscle with induced myositis [32]. FST failed to activate a large portion of muscle nociceptors with a mechanosensitive profile. The strong response to the muscle pinch suggested on the one hand that these nociceptors were nonetheless sensitised by fatiguing activity, and, on the other hand that they project on the same interneurons in the dorsal horn circuitry. Tonically active or sensitised muscle nociceptors, regardless of how they have been activated, may therefore contribute to the spinal motor control system, as has been shown for chemically activated muscle nociceptors [43]. Thus, sensitisation of the TTX-R fibres, together with tonic activation of the TTX-R muscle afferents, might be assumed as a part of the mechanism connecting chronic mechanical load and tension and chronic muscle pain.
